# Seasonal Variability of Volatilome from *Dictyota dichotoma*

**DOI:** 10.3390/molecules27093012

**Published:** 2022-05-07

**Authors:** Sanja Radman, Martina Čagalj, Vida Šimat, Igor Jerković

**Affiliations:** 1 Department of Organic Chemistry, Faculty of Chemistry and Technology, University of Split, R. Boškovića 35, 21000 Split, Croatia; sanja.radman@ktf-split.hr; 2 Department of Marine Studies, University of Split, R. Boškovića 37, 21000 Split, Croatia; mcagalj@unist.hr (M.Č.); vida@unist.hr (V.Š.)

**Keywords:** brown macroalga, volatiles, gas chromatography and mass spectrometry, pentadecane, isopachydictyol A, pachydictyol A, cembra-4,7,11,15-tetraen-3-ol

## Abstract

Dictyotaceae, the large family of brown algae with the genus *Dictyota* as the richest one among them, produce a significant number of secondary metabolites, like diterpenes. The aim of this study was to investigate the variations in the composition of the volatile organic compounds (VOCs) of the brown alga *Dictyota dichotoma* collected from the Adriatic Sea. For the first time, both seasonal changes and the impact of air drying were examined. Headspace solid-phase microextraction (HS-SPME) on two fibres with different polarities and hydrodistillation (HD), followed by gas chromatography and mass spectrometry (GC-MS) analysis, was performed on both fresh (FrDd) and air-dried (DrDd) *D. dichotoma*. The major compounds of HS-FrDd were pentadecane and oct-1-en-3-ol. The percentage of pentadecane in HS-DrDd was increased up to 7.8 times in comparison with HS-FrDd. Principal component analysis (PCA) identified differences between the variability of data among fresh and dried samples over months and clearly dissociated the fresh May samples from the others in the HS-SPME results. The most abundant group of VOCs in HD were terpenes, with diterpenes isopachydictyol A and cembra-4,7,11,15-tetraen-3-ol as the major compounds. Diterpene pachydictyol A was also found and among sesquiterpenes and gleenol was the most abundant. Based on the dominant compound analyses, the PCA showed distinct separation of the fresh and dried samples, indicating similarities between the samples and allowing the establishment of typical VOCs significant for the chemotaxonomy of *D. dichotoma*.

## 1. Introduction

The genus *Dictyota*, the richest genus of the Dictyotaceae family, contains more than 40 species, but in the Mediterranean in general, only the species *D. dichotoma* and *D. linearis* are found [1].

Brown macroalgae of the Dictyotaceae family produce a significant number of secondary metabolites, especially diterpenes, with three types of carbon skeletons: xenicanes, dolabellanes and extended sesquiterpenes including unique cyclic diterpenes [1,2,3,4]. Most of these diterpenes exhibit significant biological activities, such as antiviral, cytotoxic and chemical defensive activities [5]. Different secondary metabolites of the brown alga *D. dichotoma* were isolated and their chemical structures were elucidated including diterpenes isopachydictyolal [1], pachydictyols B and C [6], dictyols A-D, dictyol B acetate [7], dictyol-D-2β-acetate [8], dictyol E [6] and others [5]. Sulfated polysaccharides, fucose, manose, xylose and uronic acids and other fucoidans were found in *D. dichotoma* [9]. Fatty acids were also investigated in *D. dichotoma* [10]. The result of *D. dichotoma* phytochemical screening showed the presence of alkaloids, terpenoids, steroids, tannins, flavonoids, phenols, coumarins, quinones and glycosides [11]. The extracts of *D. dichotoma* from Croatia were analysed for total phenolics, flavonoids and tannins [12].

In the course of our investigations towards the bioprospecting of the Adriatic Sea and chemical profiling of secondary metabolites from different marine sources, we investigated the volatile organic compounds (VOCs)—volatilome chemical composition of the brown alga *D. dichotoma* collected from the Adriatic Sea. There are limited or scarce data on *D. dichotoma* chemical composition from the Adriatic Sea [12,13,14]. In our previous research [14], sesquiterpenes were found among *D. dichotoma* headspace VOCs, predominantly germacrene D followed by other cadinenyl (abundant), muurolenyl and amorphenyl structures. The headspace VOCs in *D. dichotoma* from the Yellow Sea of China were also characterised as sesquiterpenes (predominantly germacrene D) and diterpenes [15]. However, there is a gap in the research on less volatile compounds of *D. dichotoma* as well as about VOC seasonal variability that could be also important for the algal chemotaxonomy or biological activity. Therefore, the goals of our study are to: (a) isolate the headspace VOCs from fresh *D. dichotoma* (HS-FrDd) by headspace solid-phase microextraction (HS-SPME) and by hydrodistillation (HD; first-time research); (b) analyse VOCs by gas chromatography and mass spectrometry (GC-MS); (c) investigate the impact of air-drying (first time report) to chemical profiles of dried *D. dichotoma* obtained by HS-SPME (HS-DrDd) and HD (HD-DrDd); (d) compare the chemical profiles obtained by HS-SPME and HD within the seasons; (e) determine the variability of the VOCs composition from May to September from fresh and air-dried samples, and to elaborate the data by principal component analysis (PCA); (f) provide new insights for the algal chemotaxonomy regarding moderate and less volatile compounds and their seasonal changes, or the impact of air-drying.

## 2. Results and Discussion

The VOCs were isolated by two methods (HS-SPME and HD) and analysed by GC-MS in order to identify the full range of present VOCs (headspace, low, moderate and less volatiles compounds) in fresh and air-dried *D. dichotoma*. For HS-SPME, two fibres were used in accordance with our preliminary research, taking into account the number of identified compounds; this was conducted in order to obtain more complete chemical profiles containing a broad range of headspace compounds. Great variability among HS-FrDd volatiles was found within the months by two different fibres for HS-SPME/GC-MS: divinylbenzene/carboxen/PDMS (I) and polydimethylsiloxane/divinylbenzene (II). To collect the VOCs obtained by HD, the solvent trap was used.

### 2.1. Variations of Headspace Volatilome of Fresh D. dichotoma

The identified HS-VOCs of fresh *D. dichotoma* (HS-FrDd) within all collection months can be classified into aliphatic compounds (including C_11_-hydrocarbons (dictyopterenes)), terpenes (mainly sesquiterpenes) and C_13_-norisoprenoids. Great variability can be noted in May in comparison to other months (Table 1 and Table 2), particularly with respect to the higher abundance of sesquiterpenes. It should be noted that the sea temperature in May was the lowest (20.1 °C) in comparison with other months. Such a low temperature could affect the biosynthesis of secondary metabolites. 

The major compound of HS-FrDd for all months was pentadecane, with the highest abundance in July and August (up to 23.41% (I) and up to 11.51% (II)) and the lowest in September (4.92% (I); 2.14% (II)). Pentadecane was reported for the first time as a metabolite of *D. dichotoma* using nuclear magnetic resonance (NMR) and electron ionization mass (EIMS) spectra [2]. Its unsaturated derivative, pentadec-1-ene, was the most abundant in May (3.98% (I); 3.69% (II)). These two compounds were not present in our previous research of fresh *D. dichotoma* headspace [14], but the sample was from a different single-point collection location. On the other hand, pentadecane was found in the headspace of *D. dichotoma* from the Yellow Sea of China [15]. Another present higher alkane was heptadecane (up to 3.53% (I) or 2.35% (II)), which was not present in the research of Wang et al. [15]. Tridecanal abundance increased from May to September up to 23.24% (I) or 13.89% (II), as well as (*E*)-non-2-enal up to 1.97% (I) or 1.80% (II). (*E*)-Non-2-enal was present in *D. dichotoma* from the Yellow Sea of China [15] and tridecanal was a minor compound; several unsaturated aliphatic compounds (not present in current research) were found (e.g., heptadec-8-ene, or (*Z*)-pentadec-11-enal).

Another abundant compound from June to September was oct-1-en-3-ol (up to 19.85% (I); 27.25% (II)) with the lowest percentage in May (0.41% (I); 0.92% (II)). The same trend was noted for other major aliphatic acyclic C_8_-compounds: (*E*)-oct-2-enal (up to 5.54% (II) in June), (*E*)-oct-2-en-1-ol (up to 10.74% (I) or 15.97% (II) in September) and octan-1-ol (up to 7.42% (I) or 9.98% (II) in September). The aliphatic C_8_-compounds were found previously in HS of fresh *D. dichotoma* [14], but not in the research of Wang et al. [15]. Minor percentages of hexanal, nonanal, (*E*,*E*)-deca-2,4-dienal and pentadecanal were also periodically found (Table 1 and Table 2). Lower aliphatic compounds were not so represented in *D. dichotoma* from the Yellow Sea of China [15]; however, (*E*)-hept-2-en-1-ol was the most abundant (4.62%).

Other found aliphatic compounds were dictyopterenes: dictyopterene D’ (up to 4.42% (I) or 8.22% (II)) and periodically dictyopterene C’ (up to 0.54% (I) or 1.69% (II)). Dictyopterenes as C_11_-hydrocarbons (cyclopropanes and cycloheptadienes) and their derivatives appear to be most abundant in the brown algae of the genus *Dictyopteris* [16]. Dictyopterene C’ (also known as dictyotene) was found as a minor compound in the essential oil of vegetative parts of *Dictyopteris* [16], and also in freshly released eggs of marine brown alga *D. dichotoma*, as the substance that attracts spermatozoids [17]. Our previous research [14] also detected dyctopterenes in this alga.

The striking differences were found for HS-FrDd collected in May in comparison to other collection periods, which is particularly notable for the higher abundance of sesquiterpenes. They can be divided into the following types (Table 1 and Table 2): derivatives of cadinane and selinane followed by a minor abundance of aromadendrane, caryophyllane and other derivatives. Selinane and cadinane are the two main types of the sesquiterpenes reported in brown algae [18]. The major cadinene-type sesquiterpenes in May were: epicubenol (11.79% (I); 2.56% (II)), δ-cadinene (6.43% (I); 3.45% (II)), α-amorphene (3.40% (I); 2.51% (II)), and epi-bicyclosesquiphellandrene (2.85% (I); 2.28% (II)). Minor cadinane-type sesquiterpenes (Table 1 and Table 2) were: γ-cadinene, (*E*)-cadina-1,4-diene, τ-cadinol, α-copaene, α-cubebene, β-cubebene, γ-curcumene, γ-muurolene, α-calacorene, τ-cadinol and cubenol. The major selinene-type sesquiterpenes, also abundant in May, were germacrene D (6.11% (I); 14.39% (II)) and germacrene C (3.30% (I); 4.59% (II)), followed by γ-selinene, found periodically up to 1.94% (I) or 0.77% (II). Among aromadendrane-type sesquiterpenes, β-gurjunene (5,10-cycloaromadendrane sesquiterpene) was found in May with 5.73% (I) or 3.92% (II). α-Humulene (α-caryophyllene), as caryophyllene-type sesquiterpene, was present in May with 5.85% (I) or 3.86% (II). Among other sesquiterpenes, gleenol was present up to 1.14% (I) or 0.65% (II). Among C_13_-norisoprenoids, the most abundant was β-ionone (up to 2.45% (I) or 1.84% (II) in May), followed by β-cyclocitral.

There is a partial similarity with our previous research [14] on fresh *D. dichtyota* HS collected in the Adriatic Sea, but from another location. Namely, predominant sesquiterpene was germacrene D (28.3%) followed by cadinene-type sesquiterpenes: δ-cadinene (8.3%), γ-cadinene (3.4%), β-cadinene (2.8%) and *trans*-cadina-1,4-diene (1.2%). Additionally, β-bourbonene was identified with (5.1%) and α-copaene with a lower percentage. Compared to the VOCs results of this alga from the Yellow Sea of China [15], a lower number of sesquiterpenes were found with predominant germacrene D (34.83% with DVB/CAR/PDMS fibre; 62.00% with PDMS fibre), together with minor ones such as δ-cadinene, γ-muurolene and β-bourbonene and cubenol (up to 4.70%).

### 2.2. Variations of Headspace Volatilome of Air-Dried D. dichotoma

The predominant compound of HS-DrDd in all months was pentadecane (up to 92.83% (I) or 88.23% (II) in September). Its percentage was increased up to ca. 4.3 or 7.8 times in comparison with Fr-HSDd; this could be connected to fatty acid degradation, particularly palmitic acid, since the results of incubation of palmitic-16-^14^C acid in a culture of *Nostoc muscorum* indicated a direct decarboxylation into heptadecane and pentadecane [19], but also to the evaporation of the most volatile compounds. It is already known [20,21,22] that pentadecane predominates in the brown algae and heptadecane in the red algae, but our research demonstrates that drying could significantly influence the macroalga headspace composition, remarkably increasing the abundance of pentadecane that could be important for *D. dichotoma* chemotaxonomy.

**Figure 1:**
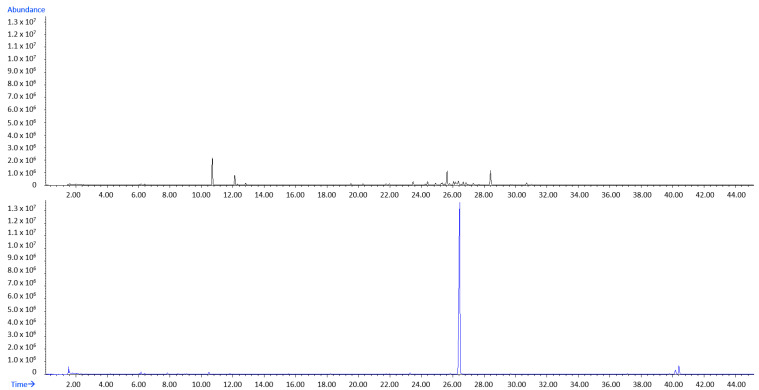
Total ion chromatogram (TIC) comparison of HS-FrDd (black) and HS-DrDd (blue) samples from May (chromatograms were zoomed to 45 min).

An array of other HS-VOCs (Table 1 and Table 2) was identified in HS-DrDd, but with less abundance including aliphatic compounds, sesquiterpenes and C_13_-norisoprenoids. Only in July and August, there was a slightly increase in other compounds and a decrease in pentadecane in HS-DrDd. In July, benzyl alcohol increased to 2.24% (I) or 2.43% (II) as well as dictyopterene D’ to 1.74% (II) and dictyopterene C’ up to 2.32% (II). Another major difference in comparison to HS-FrDd is a notable reduction in the number and abundance of sesquiterpenes in HD-DrDd that could have evaporated; this is also interesting for the chemotaxonomy. A selected example of the total ion chromatogram (TIC) from the fresh (HS-FrDd) and dried (HS-DrDd) sample is presented in Figure 1.

### 2.3. Statistical Analysis of the Headspace VOCs

**Figure 2:**
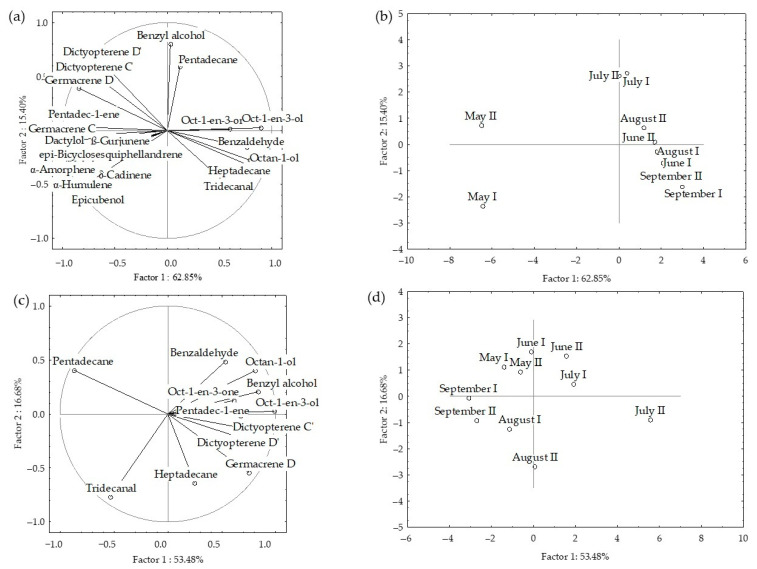
Correlation loadings (**a**,**c**) and score plots (**b**,**d**) of the dominant compounds from the headspace volatiles obtained by two different fibres for HS-SPME/GC-MS: divinylbenzene/carboxen/polydimethylsiloxane (I) and polydimethylsiloxane/divinylbenzene (II) of fresh (**a**,**b**) and dried (**c**,**d**) *D. dichotoma* samples.

**Figure 3:**
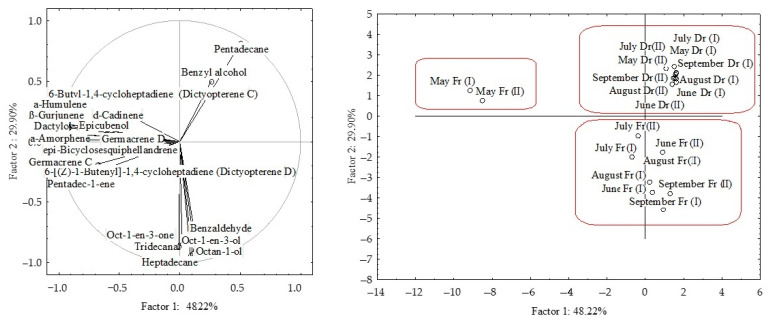
Score plot of the dominant compounds from headspace volatiles (two different fibres for HS-SPME/GC-MS: divinylbenzene/carboxen/polydimethylsiloxane (I) and polydimethylsiloxane/ divinylbenzene (II)) of fresh (Fr) and dried (Dr) *D. dichotoma* samples.

The dominant compounds (>2%) from HS-FrDd and HS-DrDd were subjected to principal component analyses (PCA) to describe the variations among the volatiles in relation to material preparation (fresh or dry) and seasonal effect. The PCA results for headspace volatiles (two different fibres) are shown in Figure 2a–d and Figure 3.

The correlation plot and score plot of the dominant components from fresh samples are shown in Figure 2a,b. The first two PCs described 78.25% of the initial data variability while the remaining PCs each accounted for less than 1% of the total variance. The correlation between certain groups of the compounds was observed (Figure 2a). The highest variable contribution, based on correlation, was observed for oct-1-en-3-ol, octan-1-ol, ß-gurjunene, α-humulene, epi-bicyclosesquiphellandrene, α-amorphene, pentadec-1-ene, germacrene C and δ-cadinene. Dictyopterene D’ and pentadecane had the highest values of factor coordinates for the PC2, with the highest variable contributions, based on the correlations. The position of the samples in the multivariate space of the first two PCs is shown in Figure 2b. Scores were arranged in two areas with a clear separation between May samples and other sampling months. The compounds that significantly affect the separation of the May sample from other months belong to the group of sesquiterpene hydrocarbons (β-gurjunene, α-humulene, epi-bicyclosesquiphellandrene, α-amorphene, germacrene D, germacrene C and δ-cadinene) and two sesquiterpene alcohols (dactylol nad epicubenol). They all strongly correlate with each other.

The correlation plot and score plot of the dominant components from dried samples are shown in Figure 2c,d. The first two PCs described 70.36% of the initial data variability. Oct-1-en-3-ol, benzyl alcohol, octan-1-ol, dictyopterene C’, and pentadecane showed the highest variable contribution to PC1 and factor-variable correlation. The July (fibre II) samples were separated in the right part of the plot based on the lowest pentadecane content, while for PC2 August sample showed the highest case contribution with the highest pentadecane content. A negative correlation was observed between pentadecane and oct-1-en-3-ol as well as pentadecane and germacrene D.

When the data were analysed together, clear separation was observed between fresh and dried samples. The dried samples appeared in the upper right corner of the plot, while the fresh ones were placed in the lower right corner (Figure 3). Based on the higher content of sesquiterpenes, sesquiterpene alcohols and pentadec-1-ene, as well as the lowest content of pentadecane, the fresh samples from May (both fibres) were segregated in the left part of the plot. There was no clear separation between the other sampling months nor correlation with temperature change over months.

### 2.4. Variations of Volatilome of Fresh D. dichotoma Obtained by Hydrodistillation

The composition of HD-VOCs (first-time report) was strikingly different in comparison to HS-VOCs from *D. dichotoma* (Table 3). The major compounds in HD-FrDd were prenylated-guaiane diterpenes isopachydictyol A (up to 19.72%) followed by its isomer pachydictyol A (up to 5.54%). They were previously isolated from several species of *Dictyota* such as *D. menstrualis*, *D. caribaea*, *D. dichotoma*, and *D. volubilis* [7,23,24,25,26,27,28]. Isopachydictyol A and pachydictyol A showed a potent antithrombotic effect through the inhibition of thrombin [27]. These compounds displayed moderate to strong cytotoxicity against hepatoma (HepG2), fibroblast (WI-38), African green monkey kidney (VERO), and MCF-7 cell lines [24]. Pachydictyol A displayed significant antifouling activity against the mussel *Limnoperna fortunei* [25]. Another abundant diterpene alcohol was cembra-4,7,11,15-tetraen-3-ol up to 33.56% in September followed by cembrene A up to 0.61% and cembrene up to 0.12%. All these compounds were not found in HS-FrDd due to their lower volatility and higher molecular masses. Cembranoids were previously detected in the Xisha soft coral *Sinularia* sp. [29], including (1*R*,3*S*)-cembra-4,7,11,15-tetraen-3-ol. In nature, cembranoids may act as chemical defence compounds against fish predators and/or compete for reef organisms, bacteria, and parasites, to ensure their protection and survival [29,30].

**Figure 4:**
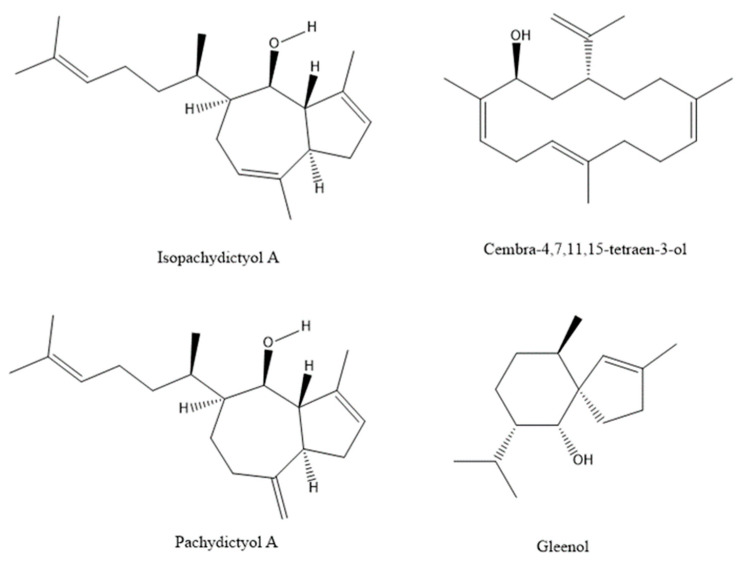
Chemical structure of most abundant terpenes in *D. dichotoma* hydrodistillate.

Among sesquiterpenes, gleenol was the most abundant up to 3.83% in May and it is known to exhibit the following biological activities: termiticidal, antihelminitic and growth regulation effects on plant seeds [31]. Other sesquiterpenes (cadinane type, selinane type, aromadendrane type and caryophyllane type) were also present (Table 3), but with a lower number and abundance in comparison to HS-FrDd. On the other hand, several sesquiterpenes were found only in HD such as (*E*)-geranyl geraniol up to 1.36%, (*Z*,*E*)-farnesyl acetate up to 1.29%; (*Z*,*E*)-farnesol up to 1.40%, caryophyllene oxide up to 0.09%, (*E*)-β-guaiene up to 0.32% or α-guaiol up to 0.26%. From C_13_-norisoprenoids, dictyopterene C’ was found up to 0.13% and β-cyclocitral up to 0.14%. The chemical structures of most characteristic compounds are presented in Figure 4.

Aliphatic C_8_-compounds were also present, particularly oct-1-en-3-one up to 2.30%, oct-1-en-3-ol up to 5.41%, (*E*)-oct-2-en-1-ol up to 3.49% and octan-1-ol up to 2.57%. However, they are less abundant in comparison to HS-FrDd.

Other present compounds were higher aliphatic ones such as methyl (all *Z*) eicosa-5,8,11,14-tetraenoate up to 11.01%, (*Z*)-octadec-9-enoic acid (up to 6.15%), tridecanal up to 5.12%, heptadecane up to 4.61% and pentadecanal up to 1.49%. Pentadecane, the most abundant compound in HS-FrDd, in HD was present periodically up to 0.28% indicating another major difference in comparison to HS-FrDd.

### 2.5. Variations of Volatilome of Air-Dried D. dichotoma Obtained by Hydrodistillation

As expected, the VOCs chemical profile of HD-DrDd was remarkably different from HS-DrDd and it showed similarity to HD-FrDd (Table 3). The major compounds were similar for HD-FrDd and prenylated-guaiane diterpenes were present: isopachydictyol A (up to 25.03%) and pachydictyol A (up to 5.12%). As in HD-FrDd, abundant diterpene alcohol was cembra-4,7,11,15-tetraen-3-ol up to 26.90% followed by cembrene A and cembrene.

**Figure 5:**
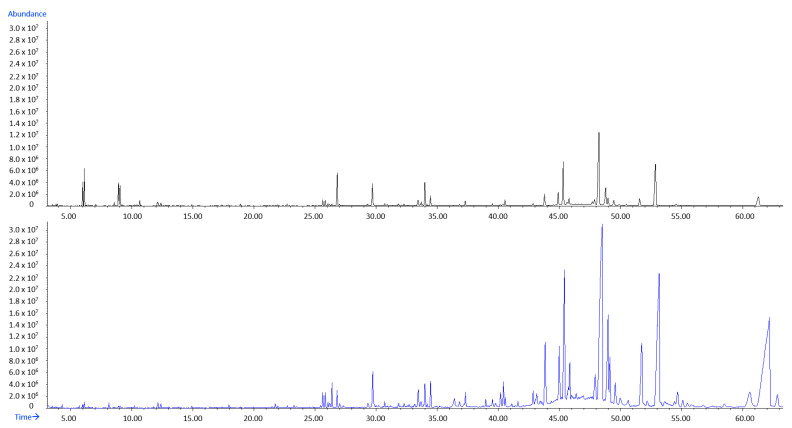
Total ion chromatogram (TIC) comparison of HD-FrDd (black) and HD-DrDd (blue) samples from May.

The most striking difference between HD-FrDd and HD-DrDd was the increase (in all months) of (*Z*)-octadec-9-enoic acid up to 11.05% and tetradecanoic acid up to 12.96%. (*Z*)-Octadecen-9-al appeared up to 4.69% in HD-DrDd. All these compounds indicate oxidation processes during drying. On the other hand, methyl (all *Z*) eicosa-5,8,11,14-tetraenoate was present in HD-FrDd and HD-DrDd. A selected example of the total ion chromatogram (TIC) from fresh (HD-FrDd) and dried (HD-DrDd) samples is presented in Figure 5.

Lower aliphatic compounds (including C_8_-compounds found in HD-FrDd) were reduced in HD-DrDd, as was expected. Only a few sesquiterpenes were found (Table 3) with germacrene D as the most abundant (up to 0.81%). Several C_13_-norisoprenoids were identified in HD-DrDd such as β-ionone (up to 1.10%), α-ionone (up to 0.14%) and β-cyclocitral (up to 0.11%) as well as periodically β-cyclohomocitral and 4-ketoisophorone.

### 2.6. Statistical Analysis of the VOCs Obtained by Hydrodistillation

**Figure 6:**
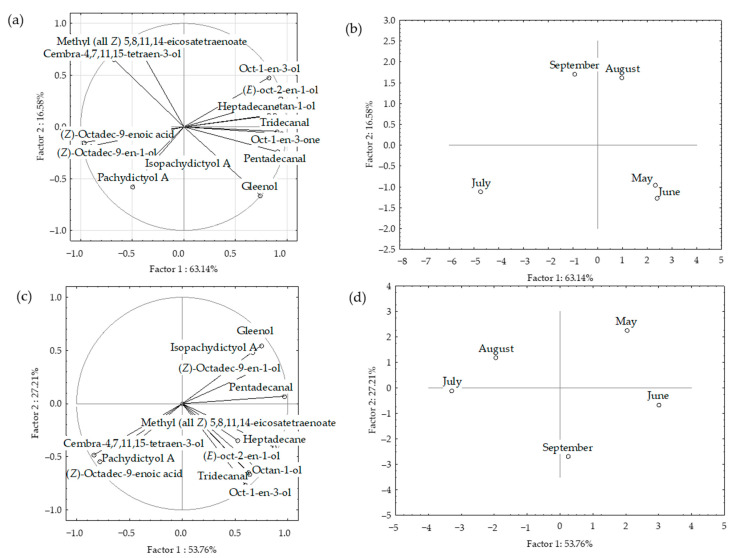
Correlation loadings (**a**,**c**) and score plot (**b**,**d**) of the dominant VOCs of fresh (**a**,**b**) and air-dried (**c**,**d**) *D. dichotoma* samples obtained by hydrodistillation.

**Figure 7:**
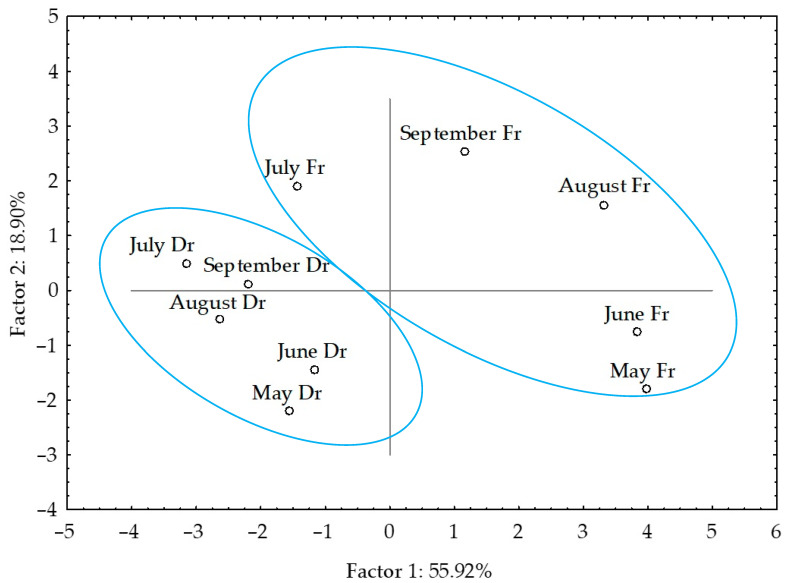
Score plot of the dominant VOCs of fresh (Fr) and air-dried (Dr) *D. dichotoma* samples obtained by hydrodistillation.

The PCA results for VOCs of fresh and air-dried *D. dichotoma* obtained by hydrodistillation are shown in Figure 6a–d and Figure 7.

In fresh samples, the first two PCs described 79.72% of the initial data variability. The correlation loadings of the first two PCs (Figure 6a) showed high correlations between two groups of compounds. Unsaturated hydrocarbons (oct-1-en-3-one, (*E*)-oct-2-en-1-ol, (*Z*)-octadec-9-en-1-ol) and higher saturated aldehydes (tridecanal, pentadecanal) were the variables with the highest variable contributions, based on the correlations. The PC2 was associated by methyl (all *Z*) eicosa-5,8,11,14-tetraenoate and pachydictyol A abundance in the samples. The score plot (Figure 6b) showed the position samples in the multivariate space of the first two PCs. Similarities were observed for May and June, and August and September, while June was segregated based on lower content of aliphatic compounds (oct-1-en-3-one, oct-1-en-3-ol, (*E*)-oct-2-en-1-ol, octan-1-ol, tridecanal, heptadecane, and pentadecanal) and diterpene alcohol gleenol, but significantly higher content of (*Z*)-octadec-9-en-1-ol, (*Z*)-octadec-9-enoic acid and diterpene alcohol, cembra-4,7,11,15-tetraen-3-ol.

In dry samples, aliphatic compounds (heptadecane, pentadecanal, (*Z*)-octadec-9-en-1-ol) and diterpene alcohol cembra-4,7,11,15-tetraen-3-ol were variables with the highest contribution to PC1, while octan-1-ol and tridecanal, contributed the most to PC2.

Oct-1-en-3-one, oct-1-en-3-ol, (*E*)-oct-2-en-1-ol and octan-1-ol positively correlated with each other, but negatively with (*Z*)-octadec-9-en-1-ol and (*Z*)-octadec-9-enoic acid. The positive correlation could be seen between saturated higher aldehydes tridecanal, heptadecane and pantadecanal, but they negatively correlated with (*Z*)-octadec-9-enoic acid.

PCA of all dominant VOCs obtained by hydrodistillation showed clear separation between fresh and dried samples (Figure 7). There were more similarities in seasonal variation between fresh samples. The dried samples appeared in the left part of the plot. The distribution was along the PC1 axis and was in the relation to aliphatic compounds (oct-1-en-3-one, oct-1-en-3-ol, (*E*)-oct-2-en-1-ol, octan-1-ol, tridecanal, and heptadecane) and (*Z*)-octadec-9-enoic acid abundance, while the distribution along the PC2 axis was related to sesquiterpene alcohols (cembra-4,7,11,15-tetraen-3-ol and gleenol) and methyl (all *Z*) eicosa-5,8,11,14-tetraenoate) abundance in the samples. Similar was observed for the fresh samples, and their vertical distribution was related to high cembra-4,7,11,15-tetraen-3-ol and low gleenol abundance. No correlation was found between the compounds’ content change over months and temperature change.

## 3. Materials and Methods

### 3.1. Sample Collection

The samples of *Dichtiota dichotoma* (Hudson) J.V. Lamouroux, 1809 were collected from May to September 2021. The location of the sampling was off the coast of the island Čiovo (43.493373° N, 16.272519° E) in the Adriatic Sea. All samples were collected from the same lagoon at a depth ranged from 20 to 120 cm. At every sampling, a YSI Pro2030 probe (Yellow Springs, OH, USA) was used to measure the sea temperature (Table 4). Marine botanist identified the alga based on its morphological characteristics. Air-drying of the samples was performed for 10 days at room temperature in shadow.

### 3.2. Headspace Solid-Phase Microextraction (HS-SPME)

HS-SPME was performed using two SPME fibres covered with DVB/CAR/PDMS (divinylbenzene/carboxen/polydimethylsiloxane) or PDMS/DVB (polydimethylsiloxane/ divinylbenzene) placed on PAL Auto Sampler System (PAL RSI 85, CTC Analytics AG, Schlieren, Switzerland). Both fibres were purchased from Agilent Technologies (Palo Alto, Santa Clara, CA, USA) and were conditioned prior to the extraction according to the manufacturer’s instructions. Prepared samples (1 g) were placed into 20 mL glass vials sealed with stainless steel cap with polytetrafluorethylene (PTFE)/silicon septa. The sample equilibration was performed at 60 °C for 15 min followed by the extraction of the sample for 45 min. Thermal desorption directly to the GC column was carried out for 6 min at 250 °C set on the injector.

### 3.3. Hydrodistillation (HD)

HD of the samples of *D. dichotoma* was performed separately for fresh (*ca.* 50 g) and dry samples (*ca.* 20 g). Modified Clevenger apparatus was used with the pentane (Fluka, Merck KGaA, Darmstadt, Germany) and diethyl ether (J.T. Baker Inc., Bridgewater, NJ, USA) in *v*/*v* ratio 1:2 (3 mL) as the solvent trap. After 2 h, the solvent trap containing dissolved VOCs was removed with a pipette, passed through the layer of MgSO_4_ in a small glass funnel and slowly concentrated by the slow flow of nitrogen until 0.2 mL. 2 µL was used for GC-MS analyses.

### 3.4. Gas Chromatography Mass Spectrometry Analysis (GC-MS)

The GC-MS analyses of isolated VOCs were carried out with Agilent Technologies (Palo Alto, Santa Clara, CA, USA) gas chromatograph model 8890 equipped with a mass spectrometer detector model 5977E MSD (Agilent Technologies). HP-5MS capillary column (30 m × 0.25 mm, 0.25 µm film thickness, Agilent Technologies, Palo Alto, Santa Clara, CA, USA) was used for the VOCs separation. The set temperatures were 250 °C for the injector and 300 °C for the detector. The oven temperature was set at 70 °C and held for 2 min followed by the temperature increment from 70 °C to 200 °C at 3 °C/min. The final temperature of 200 °C was held for 15 min. The split ratio was 1:50; carrier gas was helium (He at a flow rate of 1.0 mL/min); the MSD (EI mode) was operated at 70 eV; the mass range was set from 30 to 300 amu. The identification of the compounds was based on the comparison of their retention indices (RI), determined relative to the retention times of n-alkanes (C_9_–C_25_), with those reported in the literature (National Institute of Standards and Technology) and their mass spectra with the spectra from Wiley 9 (Wiley, New York, NY, USA) and NIST 17 (D-Gaithersburg) mass spectral libraries. The percentage composition was calculated by the normalisation method (without correction factors). The analyses were carried out in duplicate and reported as average percentage of peak area.

### 3.5. Statistical Analyses

The principal component analysis (PCA) was used to determine relations between the dominant volatiles (>2%) of fresh and dried alga samples analysed by different methods (HS-SPME and HD) [32]. The data gathered for HS-SPME from two fibres for fresh and dried samples were used for the analysis (average percentage of peak area). Similarly, for HD data gathered for fresh and dried algae samples were submitted to PCA. Prior to analyses all data were log transformed. The analyses were carried out using STATISTICA^®^ (version 13, StatSoft Inc., Tulsa, OK, USA).

## 4. Conclusions

Two methods (HS-SPME and HD) were successfully applied to identify the full range of present VOCs (headspace, low, moderate and less volatile compounds) in fresh and air-dried *D. dichotoma*. The striking differences between the profiles are not that striking considering different extraction mechanisms for HD and SPME (exhaustive distillation + solvent trap vs. partition coefficients based microextraction).

Great variability among HS-FrDd VOCs was found within the months by two different fibres. The major compound of HS-FrDd for all months was pentadecane. The great variability of HS-FrDd is noted in May in comparison to other months, particularly for the higher abundance of sesquiterpenes (derivatives of cadinane and selinane followed by a minor abundance of aromadendrane, caryophyllane and other derivatives). Since selinane and cadinane are two main types of sesquiterpenes in brown algae it is important to be aware of their seasonal variability in *D. dichotoma*. The percentage of pentadecane in HS-DrDd was increased up to *cca*. 4.3 or 7.8 times in comparison with Fr-HSDd; this could be connected to fatty acids degradation and it should be considered for *D. dichotoma* chemotaxonomy. Another major difference in comparison to HS-FrDd is the notable reduction in the number and abundance of sesquiterpenes in HD-DrDd. Based on this reduction and the changes in the abundance of dominant components over months and within the fibres used, the PCA was successfully applied to distinguish differences between the variability of data among fresh and dried samples. PCA segregated the fresh May samples from the others; however, the correlation to sea temperature changes was not observed.

The composition of HD-VOCs was strikingly different in comparison to HS-VOCs from *D. dichotoma*. The PCA showed clear separation of the fresh and dried samples based on the dominant compound analyses, indicating similarities between the samples and allowing the establishment of typical VOCs significant for the taxonomic group of *D. dichotoma*. The major compounds in HD-FrDd were diterpenes isopachydictyol A followed by its isomer pachydictyol A. Another abundant diterpene alcohol was cembra-4,7,11,15-tetraen-3-ol. Among sesquiterpenes, gleenol was the most abundant. All these compounds were not found by HS-SPME (due to their lower volatility), indicating the importance of comprehensive screening of VOCs for obtaining reliable chemical profiles for the chemotaxonomy. As expected, the VOCs chemical profile of HD-DrDd was remarkably different from HS-DrDd and it showed similarity to HD-FrDd. The major compounds were similar to HD-FrDd and prenylated-guaiane diterpenes were present. The most striking difference among HD-FrDd and HD-DrDd was the increase (in all months) of (*Z*)-octadec-9-enoic acid and tetradecanoic acid up and the appearance of (*Z*)-octadecen-9-al in HD-DrDd. All these compounds indicate oxidation processes during drying.

## Figures and Tables

**Table 1:** The volatile compounds from *D. dichotoma* isolated by headspace solid-phase microextraction (HS-SPME) and analysed by gas chromatography–mass spectrometry (GC-MS) extracted by DVB/CAR/PDMS fibre (I)–Fr (fresh), Dr (dry).

No.	Compound	RI	May		June		July		August		September	
			Fr	Dr	Fr	Dr	Fr	Dr	Fr	Dr	Fr	Dr
			Area (%)									
1	Pentanal ^a^	<900	-	-	-	0.20	-	0.12	-	0.06	-	0.07
2	Pent-1-en-ol	<900	-	0.07	-	0.21	-	-	-	-	-	-
3	Pentan-1-ol ^a^	<900	-	-	0.38	0.12	0.18	-	-	-	0.89	
4	Hexanal ^a^	<900	-	0.05	-	0.16	-	0.17	-	0.13	0.52	0.06
5	Hexan-1-ol ^a^	<900	-	-	-	-	0.32	-	0.44	-	0.39	-
6	Heptanal ^a^	907	-	0.08	-	0.15	-	-	-	0.15	-	-
7	Benzaldehyde ^a^	970	0.25	0.13	4.88	1.22	3.28	0.61	2.49	0.27	3.21	0.15
8	Oct-1-en-3-one	979	0.22	0.16	6.36	0.28	3.78	0.22	4.19	0.11	3.89	0.16
9	Oct-1-en-3-ol	985	0.41	0.51	16.40	0.89	12.03	1.26	15.87	0.62	19.85	0.07
10	Octan-3-one ^a^	991	0.55	-	0.90	-	0.55	-	1.44	-	1.20	-
11	6-Methylhept-5-en-2-one ^a^	992	-	0.24	-	0.60	-	0.26	-	0.15	-	-
12	2-Pentylfuran ^a^	996	-	0.08	-	0.13	0.30	0.14	-	0.08	-	-
13	(*E*,*E*)-Hepta-2,4-dienal	1016	-	-	-	0.09	-	-	-	-	-	-
14	Benzyl alcohol ^a^	1042	-	0.68	-	0.74	0.52	2.24	-	0.33	-	0.33
15	(*E*)-β-Ocimene	1044	-	-	-	-	0.81	-	-	-	-	-
16	Phenylacetaldehyde ^a^	1052	-	0.22	0.99	-	0.97	0.70	1.20	-	0.56	-
17	(*E*)-Oct-3-en-1-ol	1056	-	-	0.52	-	-	-	-	-	-	-
18	γ-Caprolactone ^a^	1062	-	-	-	0.30	-	-	-	-	-	-
19	(*E)*-Oct-2-en-1-al	1064	-	-	2.10	0.14	1.20	0.31	1.26	0.22	4.38	-
20	γ-Terpinene ^a^	1066	-	-	-	-	0.31	-	-	-	-	-
21	(*Z*)-Oct-2-en-1-ol	1073	-	0.16	6.96	0.24	6.53	0.63	7.49	0.14	10.74	0.05
22	Octan-1-ol ^a^	1076	0.46	0.20	4.62	0.23	3.29	0.26	4.27	-	7.42	-
23	Nonanal ^a^	1108	-	0.06	0.71	0.16	0.72	0.35	-	0.44	1.46	0.16
24	2,6-Dimethylcyclohexan-1-ol	1114	-	0.45	1.24	0.43	0.61	0.29	0.63	0.07	0.35	-
25	4-Ketoisophorone ^a^	1151	-	0.20	-	-	-	-	-	-	-	-
26	6-[(1*Z*)-butenyl]-1,4-cycloheptadiene] (Dictyopterene D’)	1158	3.01	-	0.46	-	4.42	-	1.57	-	0.62	-
27	(*E*)-Non-2-enal	1165	-	-	1.32	-	1.27	0.30	0.76	0.19	1.97	0.09
28	[6-Butyl-1,4-cycloheptadiene] (Dictyopterene C’)	1175	0.46	-	-	-	0.54	1.03	-	-	-	-
29	β-Cyclocitral	1226	-	0.10	0.72	0.16	0.38	0.22	0.47	-	-	-
30	Tridecane ^a^	1300	-	0.10		0.21	-	0.20	-	0.14	-	0.09
31	(*E*,*E*)-Deca-2,4-dienal	1320	-	-	0.89	-	-	-	-	-	-	-
32	α-Cubebene	1355	1.98	-	0.15	-	0.41	-	0.30	-	-	-
33	α-Copaene ^a^	1376	0.23	-	-	-	-	-	-	-	-	-
34	β-Bourbonene	1388	0.63	0.12	0.57	0.28	1.45	0.44	0.95	0.14	0.38	0.06
35	β-Cubebene	1393	1.21	-	0.88	-	0.44	-	0.22	-	0.16	-
36	Tetradecane ^a^	1400	-	0.10	-	0.09	-	-	-	0.11	-	-
37	β-Gurjunene	1432	5.73	-	-	-	0.50	-	-	-	-	-
38	α-Humulene ^a^	1455	5.85	-	-	-	-	-	-	-	-	-
39	epi- Bicyclosesquiphellandrene	1468	2.85	-	0.42	-	0.47	-	0.45	-	0.16	-
40	γ-Curcumene	1476	1.80	-	-	-	-	-	-	-	-	-
41	α-Amorphene	1478	3.40	-	0.44	-	0.28	-	0.28	-	0.03	-
42	γ-Muurolene	1481	1.39	0.03	0.57	0.10	1.32	0.23	0.63	0.12	0.16	0.08
43	Germacrene D	1485	6.11	0.09	1.82	0.19	6.35	0.67	2.68	0.45	0.67	0.10
44	(*E*)-β-Ionone	1490	2.45	0.46	1.87	0.47	0.71	0.67	0.93	0.40	0.54	0.17
45	Pentadec-1-ene	1495	3.98	0.10	2.03	0.15	2.47	0.16	1.53	0.17	0.92	0.09
46	Germacrene C	1498	3.30	-	0.51	-	1.06	-	1.04	-	0.48	-
47	Pentadecane ^a^	1500	7.57	85.08	9.26	83.56	23.41	74.06	16.78	75.42	4.92	92.83
48	δ-Cadinene ^a^	1511	6.43	-	-	-	-	-	-	-	-	-
49	Tridecanal ^a^	1514	3.12	0.07	6.73	0.22	4.57	0.19	11.08	0.96	23.24	0.77
50	γ-Cadinene	1518	1.35	0.17	1.67	0.19	1.45	0.32	2.19	0.36	1.08	0.18
51	(*E*)-Calamenene	1528	3.27	0.10	0.79	0.14	1.55	0.34	0.85	0.16	0.18	0.07
52	γ-Selinene	1530	1.03	-	1.44	-	1.94	-	1.23	-	0.27	-
53	Dihydroactinidiolide	1533	-	0.16	-	0.15	-	0.20	-	0.21	-	0.10
54	(*E*)-Cadina-1,4-diene	1537	1.09	-	0.32	-	0.42	-	0.40	-	0.16	-
55	α-Calacorene	1548	0.58	-	0.36	-	0.29	-	0.28	-	0.09	-
56	Dactylol	1557	8.10	-	0.16	-	0.04	-	0.18	-	0.04	-
57	Germacrene-4-ol	1578	0.84	-	1.29	-	0.09	-	0.08	-	0.06	-
58	Gleenol	1589	0.71	0.34	1.14	0.17	0.79	0.21	0.54	0.14	0.40	-
59	Epicubenol	1616	11.79	-	0.56	-	0.18	-	0.43	-	0.87	-
60	τ-Cadinol	1630	0.33	-	0.09	-	0.05	-	0.08	-	0.06	-
61	Cubenol	1647	0.45	-	0.45	-	0.28	-	0.34	-	0.16	-
62	Heptadecane ^a^	1700	0.18	0.16	2.51	0.15	1.62	0.29	3.53	0.28	3.42	0.19
63	Pentadecanal ^a^	1718	0.15	-	1.14	-	0.88	-	1.87	-	1.90	-
64	Isopachydictyol A	2127	0.92	-	3.55	-	1.19	-	1.79	-	0.34	-

^a—^identification confirmed by standard compound; other compounds were tentatively identified; GC-MS analyses were performed on HP-5MS capillary column (30 m × 0.25 mm, 0.25 µm film thickness, Agilent Technologies, Palo Alto, Santa Clara, CA, USA).

**Table 2:** The volatile compounds from *D. dichotoma* isolated by headspace solid-phase microextraction (HS-SPME) and analysed by gas chromatography–mass spectrometry (GC-MS) extracted by PDMS/DVB fibre (II)–Fr (fresh), Dr (dry).

No.	Compound	RI	May		June		July		August		September	
			Fr	Dr	Fr	Dr	Fr	Dr	Fr	Dr	Fr	Dr
			Area (%)									
1	Dimethyl sulfide	<900	-	-	-	0.29	-	2.09	-	1.03	-	3.14
2	Pentanal ^a^	<900	-	-	-	0.29	-	0.31	-	0.13	-	0.08
3	Pent-1-en-3-ol	<900	-	0.07	-	0.27	-	0.10	-	0.05	-	0.12
4	(*Z*)-Pent-2-en-1-ol	<900	-	-	0.71	-	0.29	-	0.20	-	0.43	-
5	Hexanal ^a^	<900	-	0.05	0.53	0.20	0.41	0.32	0.19	0.27	0.31	0.09
6	3-Methylbutanoic acid	<900	-	0.10	-	1.73	-	0.30	-	0.31	-	0.37
7	2-Methylbutanoic acid	<900	-	-	-	0.41	-	-	-	-	-	-
8	Hexan-1-ol ^a^	<900	-	-	-	-	0.37	-	0.52	-	0.47	-
9	Heptanal ^a^	907	-	-	-	0.16	-	0.30	-	0.36	-	-
10	Benzaldehyde ^a^	970	0.20	0.06	2.04	1.11	1.31	0.67	0.99	0.23	4.00	0.13
11	1,3,5-Trimethylbenzene ^a^ (Mesitylene)	976	-	-	0.23	-	0.16	-	0.16	-	0.24	-
12	Oct-1-en-3-one	980	-	0.21	-	0.53	-	0.37	-	0.13	-	0.06
13	3,5,5-Trimethylhex-2-ene	980	0.43	-	9.33	-	5.06	-	6.35	-	5.99	-
14	Oct-1-en-3-ol	984	0.92	0.65	27.25	1.36	17.59	1.95	23.19	0.93	24.37	0.27
15	Octan-3-one	991	0.59	-	2.00	-	0.98	-	2.89	-	0.97	-
16	6-Methylhept-5-en-2-one ^a^	992	-	0.29	-	0.93	-	0.50	-	0.22	-	-
17	2-Pentylfuran ^a^	996	-	0.06	0.63	0.23	0.76	0.34	0.33	0.18	0.26	-
18	Octanal ^a^	1007	-	-	-	-	0.25	-	-	-	-	-
19	(*E*,*E*)-Hepta-2,4-dienal	1017	-	0.03	0.33	0.11	0.21	0.16	-	0.11	0.77	-
20	*p*-Cymene ^a^	1032	-	-	-	-	0.21	-	-	-	-	-
21	(*E*)-3-Ethyl-2-methylhexa-1,3-diene	1040	-	-	0.91	-	0.44	-	0.65	-	0.66	-
22	Benzyl alcohol ^a^	1041	-	0.56	-	0.77	0.40	2.43	-	-	-	-
23	(*E*)-β-Ocimene	1044	-	-	-	-	1.98	-	-	-	-	-
24	Phenylacetaldehyde ^a^	1052	-	0.05	1.00	0.35	0.96	1.01	1.11	0.45	0.78	0.38
25	(*E*)-Oct-3-en-1-ol	1056	-	-	0.40	-	-	-	-	-	-	-
26	γ-Caprolactone ^a^ (5-Ethyloxolan-2-one)	1061	-	0.22	-	-	-	-	-	-	-	-
27	(*E*)-Oct-2-enal	1065	0.28	0.06	5.54	0.24	4.06	0.43	3.13	0.32	2.51	-
28	(*E*)-Oct-2-en-1-ol	1073	0.19	0.19	6.93	0.25	6.53	1.02	8.35	0.24	15.97	0.06
29	Octan-1-ol ^a^	1076	0.47	0.24	3.92	0.27	3.00	0.33	4.21	0.17	9.98	0.04
30	(*E*,*Z*)-Octa-3,5-dien-2-one	1098	-	-	1.80	-	-	-	-	-	-	-
31	Nonanal ^a^	1108	0.20	0.07	1.51	0.11	1.40	0.57	0.51	0.81	1.39	0.18
32	2,6-Dimethylcyclohexan-1-ol	1114	-	0.66	1.37	0.70	0.50	0.46	0.50	0.16	0.57	0.04
33	Oct-1-en-3-yl acetate	1116	-	-	0.60	-	0.25	-	0.60	-	0.37	-
34	4-Ketoisophorone ^a^	1150	-	0.16	-	0.08	-	-	-	-	-	-
35	6-[(1*Z*)-Butenyl]-1,4-cycloheptadiene] (Dictyopterene D’)	1158	8.22	0.07	1.53	0.09	7.21	1.74	2.77	0.11	0.63	0.10
36	(*Z*)-Non-2-enal	1165	-	-	1.80	-	1.70	0.32	1.05	0.24	1.63	0.10
37	[6-Butyl-1,4-cycloheptadiene] (Dictyopterene C’)	1175	1.69	-	-	-	0.98	2.32	0.76	-	-	-
38	(*Z*)-Dec-4-enal	1197	-	0.06	-	0.18	-	0.20	-	0.23	-	0.07
39	β-Cyclocitral	1226	-	0.11	0.55	0.20	0.28	0.29	0.34	0.13	0.51	0.04
40	Tridecane ^a^	1300	-	0.21	-	0.45	-	0.25	-	0.23	-	0.09
41	(*E*,*E*)-Deca-2,4-dienal	1321	-	-	0.42	-	-	-	-	-	-	-
42	α-Cubebene	1355	1.56	0.07	-	0.08	-	0.12	-	0.05	-	0.00
43	β-Bourbonene	1389	1.65	0.18	0.41	0.50	1.50	0.81	0.97	0.41		0.04
44	β-Cubebene	1394	1.82	0.20	-	-	0.24	-	-	-	-	-
45	Tetradec-1-ene	1395	-	0.10	-	0.26	-	0.30	-	0.22	-	0.05
46	Tetradecane ^a^	1400	-	0.15	-	0.12	-	0.16	-	0.12	-	0.07
47	β-Gurjunene	1432	3.92	-	-	-	-	-	-	-	-	-
48	β-Cubebene	1434	-	-	-	-	0.28	-	-	-	-	-
49	α-Humulene ^a^	1455	3.86	-	-	-	-	-	-	-	-	-
50	β-Farnesene	1462	0.18	-	-	-	-	-	-	-	-	-
51	epi-Bicyclosesquiphellandrene	1468	2.28	-	-	-	-	-	-	-	-	-
52	γ-Curcumene	1476	1.79	-	-	-	-	-	-	-	-	-
53	α-Amorphene	1478	2.51	-	-	-	-	-	-	-	-	-
54	γ-Muurolene	1481	1.07	-	-	-	0.57	0.21	0.37	0.15	-	-
55	Germacrene D	1485	14.39	-	1.33	0.27	5.48	0.82	2.95	0.67	0.56	0.11
57	(*E*)-β-Ionone	1490	1.84	0.43	0.69	0.44	0.38	0.49	0.56	0.36	0.41	0.20
58	Pentadec-1-ene	1495	3.69	0.14	1.10	0.16	1.45	0.16	1.36	0.15	0.38	0.06
59	Germacrene C	1498	4.59	-	-	-	0.64	-	0.80	-	0.27	-
60	Pentadecane ^a^	1500	5.20	84.97	9.15	79.15	9.13	62.41	11.51	72.00	2.14	88.23
61	δ-Cadinene ^a^	1511	3.45	-	-	-	-	-	-	-	-	-
62	Tridecanal ^a^	1514	2.81	-	3.76	-	3.60	-	7.01	1.04	13.89	0.74
63	γ-Cadinene	1518	1.20	-	0.70	-	0.80	-	1.41	0.30	0.77	-
64	(*E*)-Calamenene	1528	2.04	0.23	0.27	-	0.56	0.17	0.28	0.14	0.21	-
65	γ-Selinene	1530	0.77	-	0.15	-	0.04	-	0.56	-	0.46	-
66	Dihydroactinidiolide	1533	-	0.10	-	-	-	-	-	-	-	-
67	(*E*)-Cadina-1,4-diene	1537	0.89	-	0.20	-	0.16	-	0.17	-	0.37	-
68	α-Calacorene	1552	0.19	-	0.05	-	0.07	-	-	-	-	-
69	Dactylol	1557	15.38	-	0.11	-	0.04	-	0.04	-	0.05	-
70	Germacrene-4-ol	1578	0.34	-	0.20	-	0.04	-	0.06	-	0.06	-
71	Gleenol	1589	0.58	0.26	0.65	-	0.24	0.18	0.27	-	-	-
72	Hexadecane ^a^	1600	-	0.08	-	0.08	-	0.21	-	0.13	-	0.07
73	Epicubenol	1616	2.56	-	0.30	-	0.09	-	0.22	-	0.22	-
74	τ-Cadinol	1630	0.22	-	0.10	-	-	-	-	-	-	-
75	Cubenol	1647	0.21	-	0.18	-	0.09	-	2.18	-	0.10	-
76	Heptadecane ^a^	1700	0.14	0.14	0.91	0.13	0.86	0.21	1.89	0.37	2.35	0.22
77	Pentadecanal ^a^	1718	0.12	-	0.41	-	0.43	-	0.87	-	1.36	-
78	Isopachydictyol A	2127	0.34	-	0.71	-	0.31	-	0.57	-	-	-

^a—^identification confirmed by standard compound; other compounds were tentatively identified; GC-MS analyses were performed on HP-5MS capillary column (30 m × 0.25 mm, 0.25 µm film thickness, Agilent Technologies, Palo Alto, Santa Clara, CA, USA).

**Table 3:** The volatile compounds from *D. dichotoma* isolated by hydrodistillation (HD) and analysed by gas chromatography–mass spectrometry (GC-MS)–Fr (fresh), Dr (dry).

No.	Compound	RI	May		June		July		August		September	
			Fr	Dr	Fr	Dr	Fr	Dr	Fr	Dr	Fr	Dr
			Area (%)									
1	(*E*)-Hex-2-enal	<900	0.14	0.03	0.12	0.05	-	-	-	0.51	-	-
2	(*Z*)-Hex-3-en-1-ol	<900	0.01	-	0.01	-	0.01		0.08		0.10	0.05
3	Ethylbenzene	<900	0.10	-	0.08	-	-	-	-	-	-	-
4	Hexan-1-ol ^a^	<900	-	-	-	-	0.01	-	0.27	-	0.15	-
5	1,4-Xylene	<900	0.16	-	0.02	-	0.01	-	-	-	-	-
6	(*E*,*E*)-Octa-1,3,5-triene	<900	0.17	-	0.15	-	0.01	-	-	-	0.16	-
7	(*E*)-Hept-4-enal	901	0.09	0.03	0.05	0.04	-	-	-	-	-	
8	Heptanal ^a^	904	0.05	0.06	0.08	0.10	-	0.03	0.10	-	0.04	0.05
9	Benzaldehyde ^a^	968	0.05	0.06	0.06	0.09	-	0.03	0.10	0.01	0.12	0.11
10	Heptan-1-ol ^a^	974	0.06	-	0.06	-	-	-	0.07	-	-	-
11	3,5,5-Trimethylhex-2-ene	978	-	0.09	-	0.18	-	0.04	-	0.01	-	0.12
12	Oct-1-en-3-one	978	2.30	-	2.27	-	0.13	-	1.34	-	1.57	-
13	Oct-1-en-3-ol	983	3.64	0.17	5.26	0.58	0.40	0.20	5.41	0.09	4.55	0.49
14	Octan-3-one	990	0.15	-	0.22	-	0.01	-	0.21	-	0.23	-
15	2-Pentylfuran ^a^	995	0.14	0.04	0.20	0.09	0.01	0.03	0.12		0.14	0.11
16	Octan-3-ol	997	-	-	0.04	-	-	-	-	-	-	-
17	(*E*,*Z*)-Hepta-2,4-dienal	999	0.06	-	0.11	-	-	-	-	-	-	-
18	2-[(*E*)-Pent-1-enyl]furan	1005	0.07	-	0.07	-	-	-	-	-	-	-
19	(*E*,*E*)-Hepta-2,4-dienal	1014	0.15	-	0.14	-	0.02	-	0.10	-	-	-
20	Limonene ^a^	1035	-	-	-	-	-	-	0.15	-	0.07	-
21	(3*E*)-3-Ethyl-2-methylhexa-1,3-diene	1038	0.01	-	0.06	-	-	-	-	-	-	-
22	Benzyl alcohol ^a^	1042	-	-	-	-	-	-	-	-	-	0.05
23	Phenylacetaldehyde ^a^	1050	0.11	0.14	0.23	0.26	0.02	0.07	0.30	0.02	0.28	0.14
24	(*E*)-Oct-2-enal	1063	0.42	-	0.77	0.09	0.08	0.04	1.09	0.02	0.91	0.15
25	(*E*)-Oct-2-en-1-ol	1073	2.87	0.08	3.49	0.28	0.22	0.11	2.99	0.03	2.73	0.19
26	Octan-1-ol ^a^	1076	2.57	0.07	2.42	0.25	0.14	0.06	1.55	0.01	2.25	0.16
27	(*E*,*E*)-Octa-3,5-dien-2-one	1080	0.20	-	0.28	-	-	-	-	-	0.27	-
28	(*E*,*Z*)-Octa-3,5-dien-2-one	1097	0.18	-	0.24	0.21	-	-	0.14	-	0.08	0.05
29	Nonanal ^a^	1107	0.18	0.07	0.22	0.15	0.02	0.04	0.15	0.02	0.17	0.11
30	(*E*,*E*)-Octa-2,4-dienal	1113	0.19	-	0.24	0.11	0.01	0.03	0.25		0.13	0.04
31	Bicyclo [3.2.0]hept-6-ene	1120	0.69	-	0.76	-	0.55	0.25	1.39	0.04	2.07	0.20
32	2-Hydroxyisophorone (2-Hydroxy-3,5,5-trimethylcyclohex-2-en-1-one)	1122	0.10	-	0.09	-	0.02	-	0.07	-	0.08	-
33	4-Ketoisophorone ^a^	1150	-	-	-	0.05	-	-	-	-	-	-
34	(*E*,*Z*)-Nona-2,6-dienal	1158	0.90	0.17	0.78	0.32	0.21	0.12	0.66	0.04	0.79	0.26
35	(*E*)-Non-2-enal	1165	0.40	0.13	0.64	0.31	0.04	0.08	0.64	0.05	0.50	0.25
36	[6-Butyl-1,4-cycloheptadiene] (Dictyopterene C’)	1174	0.08	-	0.09	-	0.04	-	0.12	-	0.13	-
37	2,4-Dimethylbenzaldehyde ^a^	1180	0.13	-	0.17	0.09	0.03	-	0.24	-	0.11	0.08
38	3,4-Dimethylphenol	1197	-	-	-	0.06	-	0.03	-	-	-	0.07
39	β-Cyclocitral	1226	0.14	0.05	0.13	0.11	-	0.04	0.10	0.01	0.07	0.08
40	β-Cyclohomocitral	1263	0.05	-	0.07	0.10	-	0.03	-	0.02	-	0.08
41	Indole ^a^	1296	0.15	0.12	0.26	0.21	0.01	0.04	0.20	0.04	0.10	0.11
42	(*E*,*E*)-Deca-2,4-dienal	1320	0.31	-	0.47	0.10	0.02	0.04	0.23	0.01	0.11	0.08
43	β-Bourbonene	1388	0.12	0.13	0.10	0.29	0.01	0.03	0.08	0.05	-	0.08
44	β-Cubebene	1393	0.22	0.07	0.37	-	0.02	0.04	-	0.03	-	-
45	Tetradec-1-ene	1395	-	-	-	0.21	-	-	-	-	-	-
46	Dodecanal ^a^	1412	0.27	0.08	0.24	0.11	0.02	0.02	0.21	0.02	-	0.09
47	α-Ionone	1432	0.11	0.08	0.15	0.14	-	0.04	0.16	0.02	-	0.05
48	(*Z*)-Geranylacetone	1458	-	-	-	0.06	-	-	-	-	-	-
49	Alloaromadendrene	1465	0.07	-	0.11	0.07	0.02	0.03	0.24	0.02	-	0.06
50	Dodecan-1-ol ^a^	1479	0.19	-	-	-	0.02	-	-	-	-	-
51	α-Amorphene	1481	0.06	0.09	0.16	0.11	0.01	0.03	0.23	-	-	-
52	Germacrene D	1485	0.98	0.59	1.10	0.81	0.08	0.18	0.54	0.37	0.11	0.41
53	β-Ionone	1489	0.87	0.56	0.93	1.10	0.07	0.31	0.92	0.16	0.40	0.69
54	epi-Bicyclosesquiphellandrene	1495	0.36	-	0.68	-	0.03	-	0.13	-	0.05	-
55	Pentadec-1-ene	1495	-	0.22	-	0.48	-	0.14	-	0.15	-	0.18
56	(*E*)-β-Guaiene	1498	0.32	-	0.32	-	0.03	-	0.20	-	0.07	-
57	Tridecan-2-one	1499	-	0.27	-	0.47	-	0.08	-	-	-	0.14
58	Pentadecane ^a^	1500	0.27	0.87	0.28	1.08	0.15	0.05	0.16	0.09	-	0.20
59	Tridecanal ^a^	1514	4.89	0.62	4.66	1.07	0.93	0.09	5.12	0.49	1.62	1.45
60	γ-Cadinene	1520	0.37	0.22	0.52	0.38	0.04	0.07	0.47	0.12	0.21	0.24
61	β-Cadinene	1528	0.11	0.07	0.08	0.11	0.04	0.10	0.13	0.04	-	0.12
63	Dactylol	1562	0.06	-	0.11	0.07	-	-	-	-	-	-
64	Germacrene-4-ol	1580	0.29	0.27	0.44	0.45	0.02	0.05	-	-	-	-
65	Caryophyllene oxide	1587	0.05	-	0.09	-	-	-	-	-	-	-
66	Gleenol	1590	3.83	1.87	3.61	1.63	0.29	0.53	0.49	0.39	0.09	0.13
67	α-Guaiol	1592	0.26	-	0.14	-	0.02	0.03	-	-	-	-
68	Hexadecane ^a^	1600	0.22	0.17	0.27	0.20	0.04	-	-	-	-	-
69	Tetradecanal ^a^	1616	0.34	0.24	0.39	0.24	0.06	-	0.32	0.07	-	-
70	τ-Cadinol	1630	-	0.06	-	-	-	-	-	-	-	-
71	Cubenol	1647	0.31	0.19	0.41	0.26	0.07	0.10	0.50	0.13	0.16	0.21
72	α-Cadinol	1660	0.32	0.14	0.38	0.22	0.06	0.12	0.53	0.14	0.20	0.21
73	(*E*)-Heptadec-8-ene	1683	-	0.16	-	0.15	-	-	-	-	-	-
74	(*Z*,*E*)-Farnesol	1688	1.40	0.99	1.20	1.23	0.16	0.22	-	0.29	-	0.35
75	(*E*)-Heptadec-8-ene	1696	0.68	0.33	0.69	0.56	0.08	-	0.23	0.09	-	-
76	Heptadecane ^a^	1700	3.60	0.97	3.63	1.28	1.30	0.11	4.61	0.51	1.72	1.32
77	Pentadecanal ^a^	1718	1.49	0.98	1.38	0.84	0.42	0.07	1.26	0.16	0.49	0.60
78	Tetradecanoic acid ^a^	1770	0.19	0.84	0.42	1.75	0.04	4.94	-	12.96	0.26	3.93
79	(*Z*,*E*)-Farnesyl acetate	1799	0.78	0.55	0.73	0.78	0.27	0.35	1.29	0.85	0.25	0.84
80	6,10,14-Trimethylpentadecan-2-one ^a^	1850	-	0.29	-	0.18	-	-	-	-	-	-
81	Nonadec-1-ene	1897	-	0.40	-	0.39	-	-	-	0.11	-	0.16
82	Cembrene	1930	0.08	0.27	0.12	0.28	0.09	0.07	-	0.09	-	0.18
83	Palmitoleic acid ^a^	1962	-	-	-	-	-	-	-	1.23	-	-
84	Cembrene A	1969	0.42	0.81	0.59	1.07	0.44	0.85	0.61	1.17	0.35	1.00
85	Hexadecanoic acid ^a^	1975	-	1.07	-	1.32	-	0.26	-	0.54	-	-
86	Ethyl hexadecanoate ^a^	1987	-	0.49	-	0.61	-	0.43	-	1.12	-	0.54
87	(*Z*)-Octadecen-9-al	1997	-	3.93	-	4.69	-	2.25	-	1.66	-	3.15
88	Octadecanal ^a^	2025	-	-	-	0.32	-	-	-	-	-	-
89	Methyl octadecyl ether	2036	-	3.33	-	5.09	-	4.33	-	3.44	-	3.68
90	Methyl (all *Z*) eicosa-5,8,11,14, 17-pentaenoate	2040	0.34	0.21	-	-	-	-	0.22	-	0.25	-
91	Methyl (all *Z*) eicosa-5,8,11,14-tetraenoate	2045	8.97	8.20	6.51	7.49	9.73	6.95	11.01	7.28	10.31	8.70
92	(*Z*,*Z*,*Z*)-Octadeca-9,12,15-trien-1-ol	2055	1.08	0.25	1.10	-	-	-	-	-	-	-
93	(*Z*)-Octadec-9-en-1-ol	2061	1.58	0.66	1.55	0.66	4.44	-	2.43	-	1.81	-
94	(*E*)-Phytol	2116	1.05	-	-	-	-	-	-	-	-	-
95	Pachydictyol A	2120	1.86	2.25	5.19	2.60	5.54	5.12	2.09	2.04	2.93	3.67
96	Isopachydictyol A	2128	19.72	25.03	15.01	18.79	18.88	16.02	15.77	17.92	16.91	19.15
97	(*Z*)-Octadec-9-enoic acid	2147	1.90	6.61	2.85	6.28	6.15	10.81	2.71	9.08	3.73	11.05
98	(*E*)-Geranylgeraniol	2184	0.34	0.50	0.44	0.58	1.36	0.75	0.72	0.74	0.66	0.64
99	Cembra-4,7,11,15-tetraen-3-ol	2230	13.08	17.38	16.89	16.85	27.10	26.90	24.07	22.43	33.56	25.64

^a^—identification confirmed by standard compound; other compounds were tentatively identified; GC-MS analyses were performed on HP-5MS capillary column (30 m × 0.25 mm, 0.25 µm film thickness, Agilent Technologies, Palo Alto, Santa Clara, CA, USA)

**Table 4:** Changes in the sea temperature during sampling.

	May	June	July	August	September
Sea temperature (°C)	20.1	25.0	26.2	28.1	23.3

## Data Availability

Data is contained within the article.

## Sample Availability

Samples of the compounds are not available from the authors.
